# Book Review

**Published:** 1913-09

**Authors:** 


**The Practitioner's Encyclopædia of Medicine and Surgery.** Edited by J. Keogh Murphy, M.C. (Cantab.), F.R.C.S. Second Edition. London: Oxford Medical Publications. 1913.—The first edition of this book appeared in the latter half of last year, and the short period of time which elapsed before this second edition was called for is as good a testimonial as any editor could desire to the value of the work. It does not pretend to be an exhaustive manual of medicine and surgery, but is designed to provide the general practitioner with a handy book of reference. He can here find, in a succinct form, up-to-date and reliable information which will help him in the solution of very many of those medical and surgical problems which he is so constantly called upon to face in the course of the day's work. The book is divided into five parts: (1) General Medicine, (2) General Surgery, (3) Obstetrics and Gynæcology, (4) Special Regions, (5) Special Forms of Treatment. These in their turn are sub-divided into sections which treat of such varied subjects as Life Insurance Problems, Mental Disorders, and Hospital Construction. There are some one hundred and sixty contributors to the work, most of them hospital physicians and surgeons who are well known authorities upon the subjects with which they are called upon to deal in this book. Of course, with so many contributors the merits of the articles vary, but, taken as a whole, the standard of merit is high, and moreover no effort has been spared to render the information given of practical help and utility. The indexing is good, and renders reference to any subject easy. It seems to us that this book fulfils the purpose with which it was written. It cannot fail to be useful to anyone who wishes to keep his knowledge up to date, and who has neither time nor opportunity for constant reference to a medical library. We consider that any general practitioner will find in this encyclopædia a book admirably adapted to his needs, and one which will prove a valuable investment.

**Tuberculin Treatment.** By Clive Riviere, M.D., and Egbert Morland, M.D. Second Edition. Pp. xvi. 247. London: Oxford Medical Publications. 1913. 6s. net.—The first edition has been soon exhausted: in it the authors endeavoured to raise tuberculin treatment above the field of doubt and controversy, and to place its principles and practice alike on a firm basis. Since preparing the first draft four points have specially impressed themselves on the writers. Firstly, the rapid growth of institutions in the British Isles where tuberculin is given, and the safety of its careful administration. Secondly, the danger of ill-considered general reactions, and the grave doubt whether the deliberate production of a focal reaction is wise in pulmonary cases. Thirdly, the very great value of a uniform standard of measurement for tuberculin, and of the cubic millimetre as a unit. Fourthly, amongst the abundance of these new preparations the value and growing importance of the albumose-free tuberculin. The revival of tuberculin in England, under the teaching of Almroth Wright and his pupils, is now being greatly encouraged by the introduction generally of the dispensary system under the Insurance Act, where tuberculin treatment is to be in vogue, more particularly for those patients who are not able to receive sanatorium benefits. As yet it is too early to hear of any very satisfactory results, and probably there will be, to a minor degree, a repetition of the history of 1890, when it was found, after a few months' experience of the new drug, that its action was "to promote the formation of cavities and to lead to extension of the disease," "to exhaust the patient, and to cause loss of weight and strength." The authors inculcate every care and precaution, and this book will do much to prevent any recurrence of the experience of 1890. It is to be hoped that the new class of Tuberculosis Officers will read, mark, learn and inwardly digest the teachings of this carefully compiled and reliable handbook on such debatable ground.

**Notes on the Treatment of Tuberculosis.** By John Laird, L.R.C.P.I. Pp. 85. Bristol: John Wright & Sons Ltd. 1912. 2s. 6d.—The contents of this small volume are of considerable interest to all those working with tuberculosis. The author, while not denying the importance of hyperalimentation and aerotherapy, makes a strong plea for the more extended use of medicinal remedies. In particular he advocates the prolonged administration of calcium salts, and quotes many eminent authorities in support of this view. The short section on pulmonary tuberculosis contains much that in our opinion is not sufficiently generally understood in this country, namely that the important point about this distressing disease is to recognise it in its earliest and therefore most easily curable stages, that to wait until tubercle bacilli are demonstrable in the sputum is to wait until the disease is far advanced, and that the descriptions in our text-books of early pulmonary tuberculosis are in reality descriptions of advanced disease. The author also suggests that all doubtful cases should be treated as though they were definitely tuberculous.

**Diseases of the Liver, Gall-Bladder, and Bile Ducts.** By Humphry Davy Rolleston, M.A., M.D., F.R.C.P. Second Edition. Pp. xv. 811. London: Macmillan & Co. Ltd. 1912. 25s. net.—We are extremely pleased to see a second edition of this well-known book, which has been thoroughly revised and brought up to date. As a work of reference on any of the diseases with which it deals it is admirable in every way, and can be most confidently recommended to practitioners. The references to other authors throughout the text are very numerous. It is excellently illustrated with 108 figures, including many micro-photographs, in addition to seven coloured plates.

**The Medical Diseases of Children.** By T. R. C. Whipham, M.A., M.D. Oxon. Pp. xi, 417. London: University Press, 1912. 10s. 6d. net.—There is a considerable disparity between the various chapters of this work. The sections relating to development, feeding and derangements of nutrition are excellent, although we must confess some surprise that a physician who has had much practical experience of the feeding of infants should prefer lactose to the best cane sugar as an addition to the bottle. The chapters dealing with the pneumococcal, rheumatic and tuberculous infections are modelled on the latest teaching, pneumonia being rightly classed among the pneumococcal infections. We cannot understand why so important a subject in children as diseases of the blood and lymphatic glands should be treated in the same chapter as diseases of the ductless glands, nor why cretinism should be classed among diseases of the nervous system and not among diseases of the ductless glands. As some of the rarer diseases in children are briefly mentioned, we think the value of the book would be increased if references to original papers and treatises on the subject were given. We regret there is no chapter on skin diseases; we also miss any collection of recipes for the preparation of foods, or directions as to nursery hygiene. The book is excellently illustrated.

**Medical Inspection of Schools.** By Allen G. Rice, M.D. Pp. 109. Providence: Snow and Farnham Company. 1912.—This monograph is the fifty-fifth prize dissertation under the Fiske Fund. It consists of a historical and critical survey of the systems of medical inspection of elementary schools in America, Great Britain and the continent. The subject is a large one, and is dealt with by the author in a very complete and masterly way. Broad principles of organisation are clearly indicated, and there is considerable detail in the description of the various methods adopted in different countries, townships and states. Though of primary interest to doctors and administrators in the United States, there is much which will be found of value to experts in this country, as the author has devoted much space to the consideration of British school inspection, of which he is a friendly but acute critic. Judging from internal evidence, we feel that the trustees of the Fiske

Fund have awarded the prize to a thoroughly deserving essay, and we commend the book to all interested in this important subject in England.

**A Manual of Infectious Diseases Occurring in Schools.** By H. G. Armstrong and J. M. Fortescue-Brickdale, M.A., M.D. Pp. vi. 150. Bristol: John Wright & Sons Ltd. 1912. Price 3s.—This little book is issued by the Association of Preparatory Schools, for the assistance of teachers who have the care of children's health. For this purpose it is admirably fitted, and it will be found to deal not only with the commoner infectious fevers, but also with rarer school complaints, such as glandular fever, cerebro-spinal meningitis, epidemic poliomyelitis, and infectious conditions of the skin and eye. We should especially recommend to the teacher a careful study of the opening chapter, which is devoted to the consideration of the problems of infection and disinfection.

**The Nutrition of the Infant.** By Ralph Vincent, M.D. Fourth Edition. Pp. xviii., 343. London: Baillière, Tindall and Cox. 1913. 10s. 6d. net.—Dr. Vincent has added a good deal of new matter in writing this edition of this well-known book: the chapters which describe the bacteriology of milk and the intestinal disorders of infancy have been re-written to a large extent, and some excellent micro-photographs appear for the first time. The fact that a fourth edition has been called for speaks for itself, and we need say no more on behalf of the book than that it is an admirable presentation of the case for scientific substitute feeding. It must be read, not as a text-book, but as a tract in defence of certain views held by the author. We know of no book which exposes the vices of certain proprietary foods more fearlessly than this one.

**How to Diagnose Smallpox.** By W. McC. Wanklyn, B.A., M.R.C.S., M.R.C.P., D.P.H. Pp. vii, 104. London: Smith, Elder & Co. 1913. 3s. 6d. net.—The increasing neglect of vaccination justifies renewed attention to the diagnosis of smallpox, and during the last year or two several well-qualified observers have furnished guides to aid in this difficult and important matter. Dr. Ricketts' well-known book reviewed in detail the complete question, and has become a classic. Dr. Hanna furnished illustrative details of his experience, showing especially the value of vaccination and revaccination, and now Dr. Wanklyn, from his unusual opportunities as a former Medical Superintendent of the River Ambulance Service (Smallpox) of the Metropolitan Asylums Board, which gave him the supervision for diagnostic purposes of some 10,000 cases, furnishes a modest but practical compendium, based upon the teaching of Ricketts, which should prove more than useful to those for whose especial instruction it is published. In our opinion the book would hardly serve as a first text-book on the subject, but having gained the knowledge usually furnished in the text-book the practitioner is here warned of the pitfalls which may occur to all dealing with smallpox, and especially to the over-confident. It resembles a practical laboratory hand-book, in that its greatest use will be felt when in the presence of the actual experiment.

**Studies in Small-pox and Vaccination.** By WILLIAM HANNA, M.A., M.D., D.P.H. Pp. 52. Five charts, twenty-five plates. Bristol: John Wright & Sons Ltd. 1913. 7s. 6d. net.—We have been much interested in reading Dr. Hanna's new presentation of an old story, and especially in the good photographs and graphic charts which liberally adorn the volume. The "observations on the interaction of concurrent Variola and Vaccinia" (Part III) are particularly worth study, and we do not know of any work in a small compass that will furnish more cogent argument and illustration in support of the protective influence of vaccination. A very useful book, well produced and illustrated.

